# Hand-Book of Diseases of the Skin

**Published:** 1885-07

**Authors:** 


Hand-Book of Diseases of the Skin. *Edited by* H. V. Ziemssen, m. d., *Professor of Clinical Medicine in Munich, Editor of Ziemssen's Cyclopædia of the Practice of Medicine. Contributors:* Prof. H. Auspitz, m. d., *Vienna;* V. Babes, m. d., *Budapest;* Prof. E. Geber, m. d., *Klausenburg;* E. Lesser, m. d., *Leipzig;* P. Michelson, m. d., *Kœnigsberg;* Prof. A. Neisser, m. d., *Breslau;* Prof. E. Schwimmer, m. d., *Budapest;* P. G. Unna, m. d., *Hamburg;* E. Veiel, m. d., *and* Th. Veiel, m. d., *Cannstatt;* A. Weyl, m. d., *Berlin; and* Prof. H. V. Ziemssen, m. d., *Munich. Illustrated with eighty wood engravings and color prints. New York:* Wm. Wood & Company. 1885. pp. 658.

Criticism of this elegant volume, the most elaborate which has appeared in the English language since the production of Hebra's famous treatise by the Sydenham Society, is disarmed at the very outset. The American publishers state in their preface that the work is produced for free presentation to subscribers to the Cyclopædia translated by their agents. There is an old proverb relating to gifts, which is as true to-day as when it was first enunciated in the desert of Arabia.

But there is much in the present volume that will endure the severest criticism to which the best of books should be always subjected. The names of the distinguished authors who have co-operated in its production, and their active participation in some of the largest and most valuable attainments of modern pathology, gave to the original work an interest and value that will for many years insure to it the importance which it now possesses in the library of the man who devotes himself to the special study of cutaneous diseases. The translation has been remarkably well made by the American collaborators ; and where they have taken the liberty of somewhat abridging the original text, we have found by actual comparison with the other that the work has been effective in the direction of simplicity and value.

It should be remembered, moreover, that the volume is to be read and interpreted in the light of those that have preceded it. Thus, for example, the ingenious chapter on syphilis, by Neisser, is admitted to be a justifiable interpretation of the disease under discussion as one induced by the presence of bacteria,—a theory which has, if we may believe the later investigations of Lustgarten* and others, been confirmed by demonstration since the original text was printed. But the student whose knowledge of the disease was limited to the facts contained in this chapter, would be sadly ignorant of the several clinical pictures presented in course of its fullest evolution. For these, it would be necessary for him to turn to the chapters consecrated to the same subject by Bäumler in the third volume of the Cyclopædia.

If one may be permitted to criticise in any degree a work of this character, it should be purely and solely from the

*Die Syphillus-Bacillus von Dr. Sigmund Lustgarten, Assist., etc, mit 4 chromolithographirten Tafeln. Wien, 1885.

American stand-point. After patient labor, it has been at last proven, though long disputed, that the skin diseases of this country differ in the type and in the numerical preponderance of each from those seen elsewhere. For example, there have been noted during the last severe winter, in the northwestern states of this country thousands of cases of pruritus hiemalis. Numerically, this disorder has, in the region named, surpassed the skin diseases of all kinds encountered by the general practitioner. Yet the subject is dismissed in the pages of the work before us with a paragraph, Prof. Schwimmer adding that he has known "several persons" to be affected in this way in winter; while a score of pages are devoted to leprosy; and two to rhinoscleroma, no case of the latter having yet been reported by an American author of whom we have knowledge!

The same neglect, or omission, may be observed in relation to numerous contributions made by American authors to dermatological subjects of importance. The operation for permanent removal of hairs by electrolysis, for example, is dismissed with the brief conclusion that one author reports a return of fifty per cent. of such hairs after removal—a statement that on its face bears the very best proof of the value of so simple an operation, which unquestionably after patient repetition in the end accomplishes better than all other devices the result sought for. No mention is made too of Duhring's carefully studied and amply illustrated case of neuroma, reported with full pathological details, not only in his papers on that subject but also in his treatise, and yet the total number of cases of true neuroma of the skin might probably be counted on the fingers of the two hands, while the coarse illustrations of the exceedingly rare disease, given on page 613, are of "cicatricial neuroma" and "amputation neuroma" only.

The treatment of eczema of the face, exclusive of the disease as it recurs in the eyelids, nostrils, and lips, is here concluded in just five lines. It is simply astonishing to what extent book-makers in general, having a really laudable desire to cover the ground shadowed by their system of diseases, ignore the demands of practice and the urgent necessities of sufferers. Erythematous eczema of the face is, irrespective of any involvement of the nostrils, lids, and lips, one of the most formidable, distressing, persistent, and deforming of all cutaneous disorders. Its characteristics are so clearly marked that it well-nigh stands alone among the protean manifestations of the disease which it represents. A good-sized volume might well be written upon the sole subject of its management and therapy.

Yet several of the small manuals published in this country are more practical and useful guides in the treatment of this affection than the large Cyclopædia volume which comes to us heavy with the weight of German authority.

The salve-muslins, and plaster-muslins so frequently named in the volume as suitable topical applications in cutaneous diseases, are scarcely known to the general practitioner in this country, and are for many of the latter quite out of reach. We in this city have imported and employed them to some extent, but their adaptability to many patients affected with cutaneous maladies is more limited than is generally supposed to be the case; while a piece of muslin of suitable size, thickly spread with a freshly prepared salve, is often more efficacious than the imported mulls, even if carefully preserved in air-tight boxes.

Too much praise, cannot, however, be awarded the Messrs. Wood for the indefatigable pains taken in bringing out the present volume. It is a triumph of their enterprise, of their liberality, and of their wise judgment. Had the undertaking seemed formidable at the inception, it became still more so as it progressed. The original estimate of fifteen volumes of from 500 to 700 pages (upon which, by the way, the price per volume for the American work was fixed), increased at an alarming rate. New matter and material accumulated, and the authors could not be confined in their MS. to the original estimate of pages required. Almost at the start two alternatives presented themselves to the American publishers—either to increase the number of volumes, and thereby place the burden of the extra expense upon the subscribers, or to increase the number of pages in each volume, and assume the loss themselves. The publishers, with unprecedented generosity, and without even an announcement to that effect, chose the latter alternative; and so, notwithstanding that the increase amounted to upward of fifty per cent. more matter, they carried the work through to the end, assured that the benefit to their subscribers would be thoroughly appreciated by them.

While we can but believe that the work is better suited to the shelves of the library especially devoted to dermatological literature than to those of the conventional "busy practitioner," we must acknowledge our great debt to the publishers in issuing this volume, and at the same time heartily congratulate the subscribers to the American translation of V. Ziemssen's Cyclopædia on the fact that they have had presented to them such a valuable and instructive work. J. N. H.

